# Outcomes of rhomboid intercostal plane block on local anaesthesia in cardiac implantable electronic device implantation: a randomized controlled clinical trial

**DOI:** 10.1186/s12871-025-03206-8

**Published:** 2025-07-01

**Authors:** Gozde Altun, Ayla Esin, Yasemin Ozsahin, Sukru Arslan, Kerem Erkalp, Ziya Salihoglu

**Affiliations:** 1 Department of Anesthesiology and Reanimation, Istanbul University-Cerrahpasa, Institute of Cardiology, Cerrahpaşa Mah, Org. Abdurrahman Nafiz Gürman Cd. Bina no.24, 34098, Haseki Caddesi No:32, Fatih, İstanbul, 34096 Turkey; 2 Department of Cardiology, Istanbul University-Cerrahpasa, Institute of Cardiology, Cerrahpaşa Mah, Org. Abdurrahman Nafiz Gürman Cd. Bina no.24, 34098, Haseki Caddesi No:32, Fatih, İstanbul, 34096 Turkey; 3 Department of Anesthesiology and Reanimation, Istanbul University-Cerrahpasa, Cerrahpasa Medical School, Cerrahpaşa, Koca Mustafapaşa Cd. No:53, Fatih, İstanbul, Turkey

**Keywords:** Rhomboid intercostal plane block, Postoperative analgesia, Cardiac electrophysiology, Cardiac implantable electronic devices, Arrhythmia, Satisfaction

## Abstract

**Background:**

Rhomboid intercostal plane block (RIB) has been described in the last decade. In this study, we aimed to evaluate the outcomes of RIB in terms of perioperative analgesia and patient and physician satisfaction in patients undergoing CIED.

**Methods:**

The randomized controlled trial was structured into two arms, each comprising 25 patients and allocation was performed using a sealed-envelope randomization technique. Local anaesthesia group-LAG received only local anaesthesia (LA) (prilocaine 2%) as the standard procedure. RIB group-RIBG received RIB in addition to the standard procedure. Demographic and clinical data about the patients and the procedures performed were recorded. The first and second rescue treatments in cases of pain during the procedure and the first and second rescue treatments during the postoperative follow-up were also recorded. Pain levels during the procedure, at the end of the procedure and 3, 6, 12, and 24 h after the procedure were determined according to the NRS-11(Numeric Rating Scale-11). Patient and physician satisfaction were recorded on a five-point Likert scale. The primary outcome of the study was the efficacy of RIB in patients undergoing CIED implantation, assessed by NRS-11 pain scores during the procedure and in the postoperative period. As secondary outcome, patient and physician satisfaction levels were evaluated during the procedure.

**Results:**

There was less need for additional local anaesthetic in the RIBG than in the LAG (*p* < 0.001). NRS-11 scores during implantation, at the end of the implantation procedure, and at the 3-, 6-, 12-, and 24-hour follow-ups were greater in the LAG (*p* < 0.001). The satisfaction levels of both doctors and patients were higher in the RIBG (*p* < 0.001).

**Conclusions:**

RIB application in CIED implantations showed adequate analgesic efficacy in patients during the perioperative period and up to 24 h postoperatively. At the same time, it provided high procedural satisfaction for patients and physicians. RIB should be considered in the multimodal analgesia approach in the perioperative analgesia of this patient group. There is a need for collaboration between anaesthesiologists and cardiologists to establish protocols, including RIB, for perioperative pain management in patients who undergo CIED implantation.

**Trial registration:**

This study was registered in the Clinical Trials (ID: NCT06449599, 27/05/2024).

##  Introduction

The use of regional anaesthesia techniques for analgesia in thorax-related interventional procedures has increased in recent years. These methods have proven more effective and safer, especially with the increased use of ultrasonography in anaesthesia practice. Thus, the performance of different nerve and fascial plan blocks has increased [1]. The rhomboid intercostal plane block (RIB) was described by Elsharkawy et al. in 2016 and was applied to the posterior chest wall to provide hemithoracic analgesia [2]. It is effective in providing analgesia in different clinical conditions, such as breast surgery, thoracoscopic surgery, and myofascial pain syndrome [3–8].

The use of cardiac implantable electronic device (CIED) implantation is increasing due to comorbidities in the elderly population [6]. Approximately 1.5 million CIED implantations are performed annually worldwide [9]. CIEDs are effectively used to protect patients from sudden cardiac death, improve their hemodynamic status, and manage rhythm disorders [10]. CIEDs are administered through the anterior thoracic wall. The implantation site of devices such as cardiac pacemaker (CP), implantable cardioverter defibrillator (ICD) and cardiac resynchronization therapy (CRT) devices, which are types of CIED, is the right or left deltopectoral fossa [9]. Implantation is performed subcutaneously, subfascially, or subpectorally over or under the pectoralis major muscle [11]. Perioperative pain management in these patients is important for ensuring patient comfort, reducing complications, and maintaining stable hemodynamic values.

Patients who undergo CIED implantation usually have various comorbidities. Therefore, the increased myocardial stress associated with pain may increase the frequency of adverse cardiac events. Involuntary movements of the patient to avoid pain may lead to complications such as pneumothorax, arterial hematoma, bleeding, and lead malposition [12, 13].

CIED implantation is commonly performed under local anaesthesia (LA) and conscious sedation (CS) [14]. However, the fact that LA is not evenly distributed across the surgical field poses a problem. Therefore, patients may feel pain during venous puncture, especially during the creation of the ICD pocket. In addition, patients often experience pain in the postoperative period. It is known that paracetamol and/or opioid-derived drugs are frequently used as analgesic treatments in the perioperative period [15]. However, it has been demonstrated that adequate perioperative analgesia cannot be provided with intravenous drugs, and patients feel severe pain during the procedure despite the application of LA [15]. One-third of CIED patients even need opioids during the postoperative period [11]. It is also known that acute pain experienced after the CIED procedure poses a risk for chronic pain and chronic shoulder dysfunction [16].

This study aimed to evaluate the efficacy of RIB in patients undergoing CIED implantation by performing pain assessments with the Numeric Rating Scale-11 (NRS-11) during the procedure and in the postoperative period as primary outcome. As secondary outcome, patient and physician satisfaction levels were evaluated during the procedure.

## Methods

The primary outcome of the study was the efficacy of RIB in patients undergoing CIED implantation, assessed by NRS-11 pain scores during the procedure and in the postoperative period. As secondary outcome, patient and physician satisfaction levels were evaluated during the procedure.

### Study group

Ethics committee approval was obtained from the Istanbul University-Cerrahpasa Clinical Research Ethics Committee (E-83045809-977409, 8 May 2024) and the study was registered on clinicaltrials.gov with the number NCT06449599. The report of this study adheres to the Consolidated Standards of Reporting Trials (CONSORT) guidelines. The study was conducted as a prospective, single-center, randomized clinical trial in the cardiac electrophysiology laboratory of the Istanbul University-Cerrahpasa Institute of Cardiology. During the study, all the investigators adhered to the principles of the Declaration of Helsinki. Informed written consent was obtained from all patients.

Patients over 18 years of age who had not undergone previous CIED implantation, who did not have any infection in the CIED pocket and leads, who did not have bleeding diathesis, and who agreed to participate in the study and signed the consent form were included in the study.

Morbid obesity (BMI > 35 kg/m2), advanced decompensated heart failure (New York Heart Association -NYHA Stage 4), severe chronic obstructive pulmonary disease, known allergy to the drugs to be administered, infection or lesion in the region to be blocked, inability to communicate, inability to position, severe psychosis, progressive neurological deficit or muscular disease, CIED revision/upgrade, CIED battery replacement, pregnancy and refusal to participate in the study were the exclusion criteria of this study.

### Anatomical landmarks and rhomboid intercostal plane block

Cardiac implantable electronic devices include CP, ICD, and CRT devices. In transvenous placement of the CIED, the lead is inserted into the right ventricular apex via the cephalic vein, axillary vein, extrathoracic subclavian vein, and intrathoracic subclavian vein. The CIED battery is placed subcutaneously, subfascially, or subpectorally over or under the pectoralis major muscle at the T2–T3 level below the clavipectoral junction, anterior to the anterior axillary line [17–19].

The RIB is applied to the region defined as the “auscultation triangle (AT)” [20]. The lateral part of the plane’s base between the AT and the intercostal muscles extends deep to the scapula and serratus anterior muscle. The lateral cutaneous branch of each intercostal nerve pierces the internal and external intercostal muscles and crosses the mid-axillary line. The medial wall of the triangle extends deep to the erector spinae muscle to the transverse processes of the thoracic vertebrae. The dorsal branches of the thoracic intercostal nerves exit from the tip of the transverse processes of the thoracic vertebrae and contribute to the innervation of the erector spinae muscle and the skin [20]. When methylene blue was applied for RIB, it was shown that methylene blue spread in the lateral fascia in the clavipectoral region between the cranial aspect of T2 and the caudal aspect of T9 [2].

**Fig. 1:**
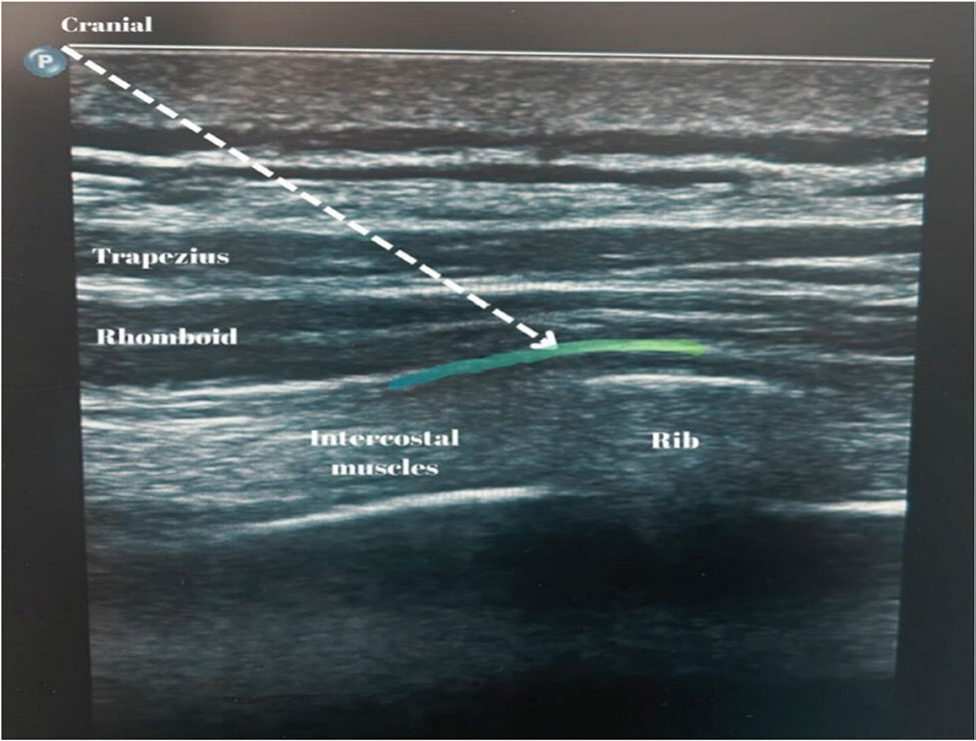
Ultrasonographic image of rhomboid intercostal plan block (RIB). A dashed line is used to illustrate the block needle, and the blue-shaded area corresponds to the intended target site for the RIB

In this study, patients undergoing RIB were monitored (noninvasive blood pressure, peripheral oxygen saturation, 5-electrode electrocardiogram monitoring) according to the American Society of Anesthesiologists (ASA). The RIB was performed 60 min before the procedure to ensure an adequate effect. In the sitting position, the scapula was abducted by bringing the arms towards the anterior chest wall. This position allowed the scapula to slide laterally. The application site was then prepared in accordance with aseptic principles. The ultrasound examination for RIB was performed with a Philips Epiq 7 ultrasound machine with a linear 12–4 MHz transducer (Philips Ultrasound; Bothell, WA, USA). The transducer was placed in the oblique‒sagittal plane from the T5 level (by counting the ribs cranially starting from T12) medial to the scapula. The trapezius, rhomboid major, and intercostal muscles were visualized (Fig. 1). A 22-gauge, 80 mm long (Echoplex+, VYGON, France) peripheral nerve block needle was passed through the skin and subcutaneous tissue to reach the fascial plane between the rhomboid major and intercostal muscles. Two millilitres of saline was used for hydrodissection to confirm the needle location. Thirty millilitres of 0.25% bupivacaine (0.5% Buvasin, 20 ml, Vemilaç, Turkey) was administered, and cranio-caudal spread was observed [21].

The cardiologist administered local anaesthesia to the puncture and battery pocket areas in all patients according to the standard procedure. As our institute’s standard practice, 20 ml of prilocaine (Priloc 2%, 20 ml, Vemilaç, Turkey) was used for LA.

After waiting for 5 min for anaesthesia onset, venous punctures were performed for the leads by targeting the axillary or extrathoracic subclavian veins. Guidewires were left at the puncture sites for sheaths. For CIED battery insertion, a 3–5 cm incision was made parallel to the clavicle, 5–10 cm below the clavicle. The skin, subcutaneous tissue, and pectoral fascia were opened via blunt dissection. A subfascial or subpectoral pocket was created via blunt dissection in the anterior plane. Subsequently, the sheaths were placed over the guidewires, and the leads were implanted. The leads were fixed to the pectoral muscle and then placed in the pocket with the battery. Deep tissues were sutured with a 0/0 silk suture, and superficial tissues were sutured with a 2/0 absorbable suture.

After the procedure, patients were transferred to the coronary intensive care unit (CICU). They were followed up for 24 h with standard monitoring and shoulder joint immobilization on the implanted side. As standard practice, venous blood gas analysis was performed after the patients were admitted to the CICU. Patients were discharged after 2 days of follow-up on the inpatient floor.

### Assessment of block success

A 60-minute interval was left after the RIB. Before the application of LA, a cold sensory loss test was performed with an alcohol-based skin disinfectant [22, 23]. The block was considered successful if patients felt less or no cold in the affected dermatomes than in the unblocked areas.

### Data collection

The study was conducted between June 2024 and January 2025. Randomization was conducted using a sealed, opaque envelope technique, with envelopes prepared and numbered in advance to maintain allocation concealment. In accordance with the study design, a randomization list for the two groups was generated by an independent individual using a computer-assisted program (www.randomizer.org). Equal numbers of participants were assigned to either the RIBG or LAG in a 1:1 ratio. The group assignment corresponding to each randomization sequence (RIBG and LAG) was written on separate sheets of paper, folded, and sealed in opaque, light-proof envelopes. It was confirmed that the contents of the envelopes could not be discerned from the outside, even when held up to light. Each envelope was labelled only with a sequential number. The envelopes were stored in numerical order. For each new patient, the next envelope in the sequence was opened, and the patient was assigned to the corresponding group based on the information inside. Throughout the entire randomization process, it was ensured that the envelopes were not opened in advance, altered, or resequenced. The patients included in the study were divided into 2 groups of 25 patients each: Local anaesthesia group (LAG) and the group that received a rhomboid intercostal plane block in addition to local anaesthesia fascial (RIBG). The LAG underwent LA as a standard procedure, and the RIBG underwent ultrasonography-guided RIB in addition to the standard procedure. Premedication was not administered to either group, in accordance with the institute’s standard procedures. CIED implantation was performed by an experienced cardiologist (> 100 cases of CIED implantation per year). RIB with ultrasonography (USG) was performed by an anaesthesiologist with at least 10 years of experience in regional anaesthesia with USG guidance.

Demographic and clinical data, including age, sex, BMI, and comorbidities, were recorded in the database. The vein (axillary, subclavian), number of leads, difficulty of venous puncture (2 or more puncture attempts were considered ‘difficult punctures’), side (right/left) of CIED insertion, and site of battery placement (subcutaneous, subfascial, subpectoral) were also recorded in the same database. The data also included the total procedure time, fluoroscopy time, and total amount of LA used. Complications during the procedure, additional local anaesthetics administered intraoperatively (first rescue treatment and second rescue treatment), and the need for postoperative analgesics (first rescue treatment and second rescue treatment) were also included in the database. When effective analgesia could not be achieved, the patient was informed about alternative methods. The outcome assessor (cardiologist) was blinded, whereas the patient was not, due to the nature of the study. The highest levels of pain were recorded during implantation, at the end of the procedure, and at 3, 6, 12, and 24 h after operation via the NRS-11 [24]. No pain was defined as “0 points,” and the worst possible pain level was defined as “10 points”. A five-point Likert scale was used for patient and physician satisfaction [25]. One point corresponded to “not at all satisfied”, and 5 points corresponded to “very satisfied”. An additional 100 mg of prilocaine was administered to the area of pain as the first-line rescue treatment in both groups if the intraoperative NRS-11 score was ≥ 4. If the patient had an NRS-11 score of ≥ 4 again during the procedure, 100 mg of prilocaine was administered again as the second rescue treatment.

In the postoperative follow-up of the patients in both groups, 1 g of paracetamol (Partemol 1 gr/100 ml, Vemilaç, Turkey) was administered intravenously as an infusion for 20 min as the first rescue treatment if the patients had an NRS-11 pain score of ≥ 4. Two hours after intravenous (IV) paracetamol administration, if the NRS-11 score was 4 or higher, 50 mg of tramadol HCL (Tradolex, 100 mg/2 ml; Mentha Pharma, Turkey) was administered intravenously as the second rescue treatment. In addition, when the NRS score was ≥ 4 at follow-up, paracetamol (1 g, IV) was administered with dosing intervals not shorter than 6 h.

### Statistical analysis

A search of academic databases, it appears that this study is the first of its kind in the field. Therefore, there is no existing article that could be used as a reference for the sample size calculation. A literature review was conducted regarding the minimum sample size required for a pilot study in a randomized controlled trial. In the study by Whitehead et al., the recommended values for five different researchers, presented in Table 4, were examined [26]. Jolious [27] suggested a sample size of 24 for pilot studies, Keiser and Wassmer [28] recommended a range of 20–40, and Browne [29] proposed a sample size of 30 for pilot studies. These pilot data meet the minimum sample size recommended by Whitehead et al. [26]. Additionally, an a priori power analysis was conducted using G*Power software (version 3.1.9.7, University of Düsseldorf, Germany) to estimate the required sample size for a chi-square goodness-of-fit test based on a 5-point Likert scale. With five response categories, the degrees of freedom were set to 4. Assuming a large effect size (w = 0.5), a significance level of 0.05, and a statistical power of 0.80, the analysis indicated that a minimum of 48 participants would be required to detect a significant deviation from the expected distribution.

Statistical analysis was performed using SPSS software (version 21.0; IBM Corp., Armonk, NY, USA). Continuous variables were reported as medians with minimum and maximum values, while categorical variables were expressed as frequencies and percentages. The normality of data distribution was assessed using the Shapiro–Wilk test. Between-group comparisons of continuous variables were conducted using the Mann–Whitney U test, and categorical variables were analyzed using the chi-square test. Pearson’s correlation analysis was used to examine associations between continuous variables. A *p*-value of < 0.05 was considered statistically significant.

## Results

**Fig. 2:**
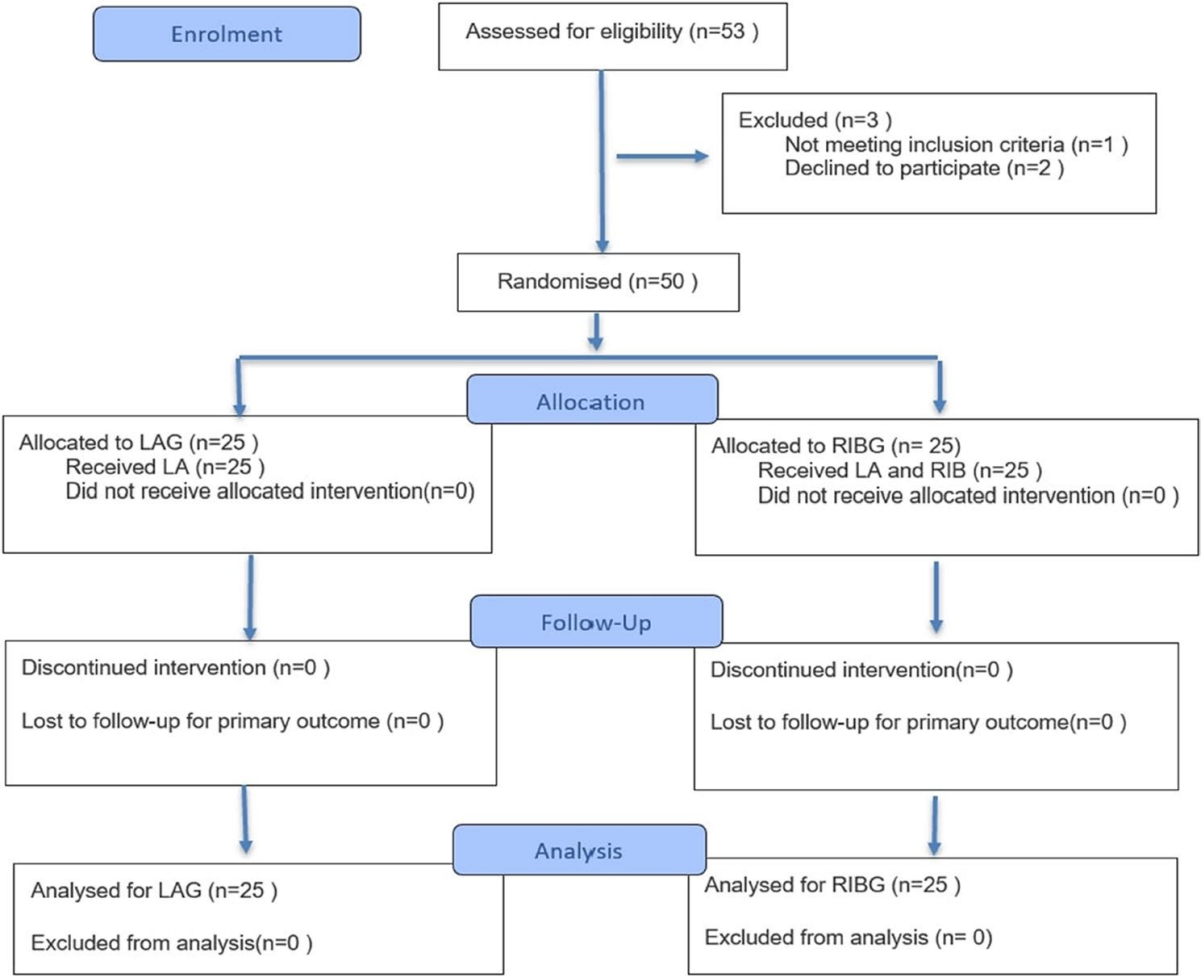
Flow chart of the study. LA: Local anaesthesia; RIB: Rhomboid intercostal plane block; LAG: Local anaesthesia group; RIBG: Rhomboid intercostal plane block group

The study was conducted in the cardiac electrophysiology laboratory of the Istanbul University-Cerrahpasa, Institute of Cardiology, between June 2024 and January 2025; with 50 patients who met the study criteria and underwent CIED implantation (Fig. 2).

The median age of the patients was 64 (42–89) in the RIBG and 68 (45–84) in the LAG. While 72% of the patients in the RIBG were male, this rate was 80% in the LAG. The demographic data of the patients are shown in Table 1.

**Table 1:** Baseline demographic characteristics of the study groups

	RIBG (*n*:25)	LAG (*n*:25)	*P* value	95% CI
Age (years)^a^	64 (42–89)	68 (45–84)	0.620	–7.00 to 10.00
Male (%)	18 (72.0)	20 (80.0)	0.508	
BMI (kg/m2)	26,91 ± 3,8	27,79 ± 3,9	0.574	–2.15 to 3.07
Hypertension, n (%)	15 (60.0)	17 (68.0)	0.556	
Diabetes Mellitus, n (%)	15 (60.0)	15 (60.0)	1.000	
Hyperlipidaemia, n (%)	2 (8.0)	5 (20.0)	0.221	
CAD, n (%)	14 (56.0)	16 (64.0)	0.564	
CKD, n (%)	5 (20.0)	3 (12.0)	0.440	
AF, n (%)	7 (28.0)	11 (44.0)	0.239	

*AF* atrial fibrillation, *BMI* body mass index, *CAD* coronary artery disease, *CKD* chronic kidney disease, *LAG* local anaesthesia group, *RIBG* rhomboid intercostal plane block group, *CI* confidence interval

^a^Median (min–max)

ICDs were the most commonly implanted CIED (Table 2). ICDs were implanted in 60% of patients in LAG and 68% in RIBG. One or two leads were implanted in both groups. In both groups, implantation was mostly performed in the left pectoral region (RIBG: 96%, LAG: 92%). The battery implantation site was mostly subpectoral in both groups (RIBG: 76%, LAG: 72%). The subclavian vein was most frequently used to implant CIEDs (RIBG: 64%, LAG: 72%).

As shown in Table 2, there was a significant difference between LAG and RIBG in the total dose of prilocaine HCl used during the procedure (*p* < 0.001). The median volume of prilocaine used was 20 ml in the RIBG and 30 ml in the LAG.

The groups were similar in terms of the total procedure times and fluoroscopy times.

**Table 2:** Procedural characteristics of the study groups

	RIBG (*n*:25)	LAG (*n*:25)	*P* value	95% CI
Device				
Pacemaker, n (%)	4 (16.0)	4 (16.0)	0.769	
ICD, n (%)	17 (68.0)	15 (60.0)		
CRT, n (%)	4 (16.0)	6 (24.0)		
Number of Lead Impl.				
One Lead, n (%)	1 (4.0)	3 (12.0)	0.497	
Two Leads, n (%)	18 (72.0)	18 (72.0)		
Three Leads, n (%)	6 (24.0)	4 (16.0)		
Left Pectoral Site Impl., n (%)	24 (96.0)	23 (92.0)	0.552	
Subpectoral Muscle Impl., n (%)	19 (76.0)	18 (72.0)	0.747	
Subclavian Vein Usage, n (%)	16 (64.0)	18 (72.0)	0.544	
Difficult Puncture, n (%)	3 (12.0)	6 (24.0)	0.269	
Amount of Prilocaine HCL (ml)^a^	20 (20–25)	30 (25–30)	**< 0.001**	**–10.00 to − 10.00**
Total Procedural Time (min)a	132 (92–238)	125 (67–248)	0.225	–11.00 to 29.00
Total Fluoroscopy Time (min)^a^	16.9 (4.6–53.0)	16.7 (3.8–56.7)	0.308	–9.68 to 4.92

Boldfaced *p*-value indicate statistically significant result

*CRT* cardiac resynchronization therapy, *ICD* implantable cardioverter defibrillator, *HCL* hydrochloride, *Impl*. implantation, *LAG* Local anaesthesia group, *RIBG* Rhomboid intercostal plane block group, *min* minutes, *ml* millilitre, *CI* confidence interval

^a^Median (min–max)

### Analysis of the use of additional local anaesthesia

A comparison of the need for additional LA during implantation revealed that the need for additional local anaesthetic was greater in LAG (*p* < 0.001) (Table 3).

**Table 3:** Analysis of supplemental local anaesthetic and analgesic use

	RIBG (*n*:25)	LAG (*n*:25)	*P* value
**Local anaesthetic supplementation**,** n (%)**	1 (4%)	25 (100%)	**< 0.001**
**First rescue analgesia**,** n (%)**	1 (4%)	24 (96%)	**< 0.001**
**Second rescue analgesia**,** n (%)**	0 (0%)	8 (32%)	**0.002**

Boldfaced *p*-values indicate statistically significant results

*LAG *Local anaesthesia group, *RIBG *Rhomboid intercostal plane block group

### Analysis of pain scores

**Fig. 3:**
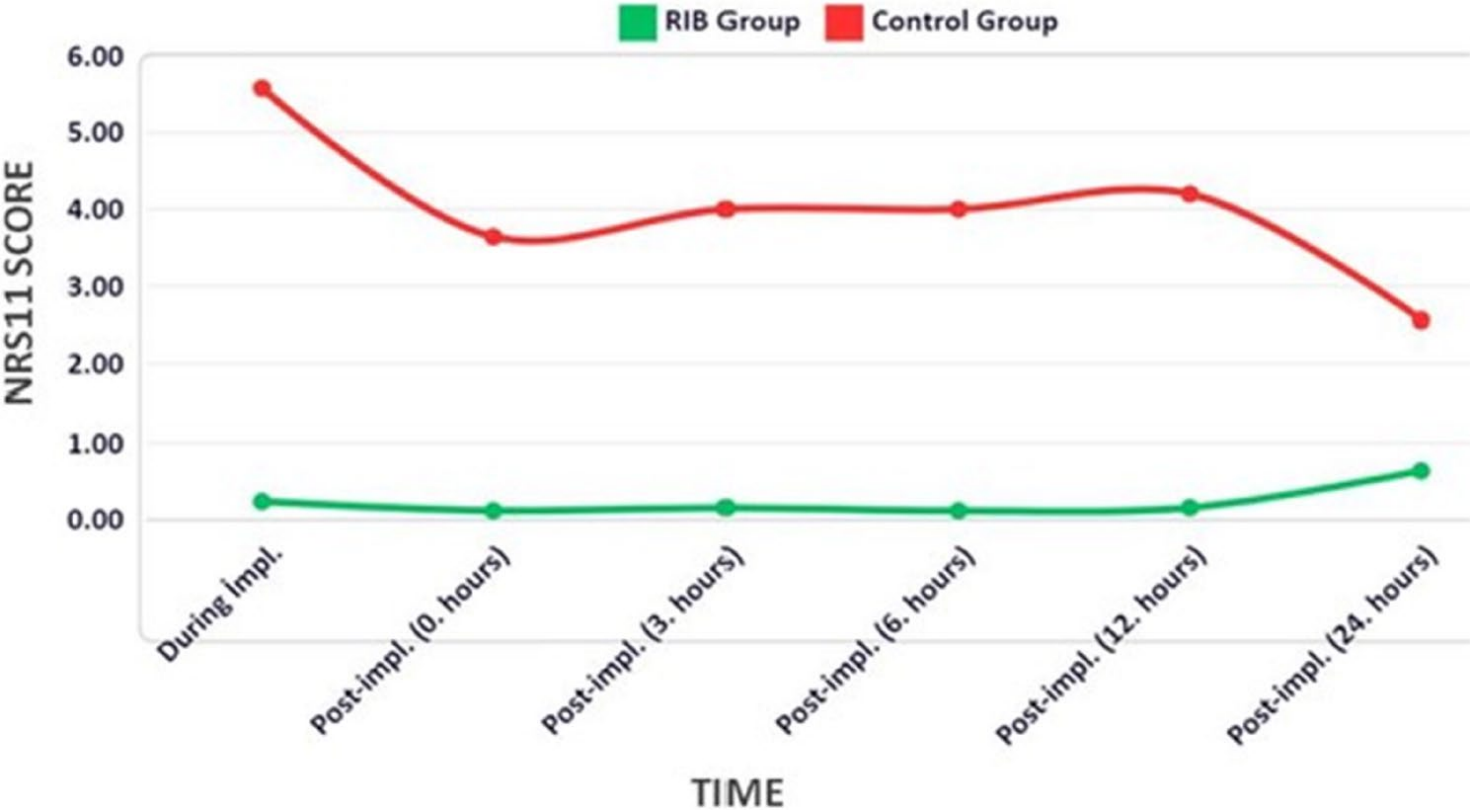
Changes in NRS-11 mean scores over time. Impl: implantation

NRS-11 scores were used to assess pain at the time of implantation, at the end of implantation and at 3, 6, 12, and 24 h after implantation. In the RIBG, the median scores were consistently 0 at all measured intervals, including the intraoperative and postoperative periods (Table 4). In LAG, the highest median value was 6, indicating that pain was felt during implantation (Fig. 3).

A statistically significant difference was found between LAG and RIBG in favour of RIBG (*p* < 0.001) (Table 4), both in terms of pain during implantation at the end of the implantation procedure and at the 3rd, 6th, 12th, and 24th hours of follow-up.

**Table 4:** Procedural characteristics of the study groups

	RIBG (*n*:25)	LAG (*n*:25)	*P* value	95% CI
NRS-11				
Procedural period^a^	0 (0–2)	6 (2–8)	**< 0.001**	**−5.0 to −3.0**
Postprocedural immediate^a^	0 (0–1)	3 (0–7)	**< 0.001**	**−5.0 to −3.0**
Postprocedural 3. hours^a^	0 (0–2)	3 (2–9)	**< 0.001**	**−3.0 to −1.0**
Postprocedural 6. hours^a^	0 (0–1)	3 (0–7)	**< 0.001**	**−6.0 to −3.0**
Postprocedural 12. hours^a^	0 (0–2)	3 (1–7)	**< 0.001**	**−4.0 to −1.0**
Postprocedural 24. hours^a^	0 (0–3)	2 (0–6)	**< 0.001**	**−1.5 to −0.5**
Likert Scale (doctor)^a^	5 (4–5)	3 (1–4)	**< 0.001**	**1.0 to 2.0**
Likert Scale (patients)^a^	5 (4–5)	3 (2–4)	**< 0.001**	**2.0 to 3.0**

Boldfaced *p*-values indicate statistically significant results

*SD s*tandard deviation, *LAG *Local anaesthesia group, *RIBG *Rhomboid intercostal plane block group, *CI *confidence interval

^a^Median (min–max)

Paracetamol administration was planned as the first rescue treatment in patients with an NRS-11 score ≥ 4 at the postoperative follow-up. When comparing LAG and RIBG in this regard, the need for the first rescue treatment was observed in only 1 patient (4%) in the RIBG, whereas 96% of patients in the LAG required it. (*p* < 0.001).

In the postoperative follow-up of the patients, tramadol HCl, an opioid derivative, was administered if the NRS-11 score was ≥ 4 2 h after the first rescue treatment. When LAG and RIBG were compared, the need for a second rescue treatment was observed in 32% of patients in LAG, whereas none of the patients (0%) in RIBG needed this treatment. There was a significant difference in the need for opioids during the postoperative period between the two groups (*p* < 0.002) (Table 3).

### Analysis of patient and physician satisfaction levels

As a result of the comparative analysis of the satisfaction levels of patients and physicians both during implantation and 24 h postoperatively, according to the Likert scale, both patient and physician satisfaction levels were higher in the RIBG (*p* < 0.001) (Table 4).

In the correlation analysis, there was a strong (*r* = 0.857) positive correlation between patient and physician satisfaction (*p* < 0.001). Additionally, there was a strong negative correlation between pain during implantation and patient and physician satisfaction (*p* < 0.001). There was a strong negative correlation (*r*= −0.749) between the NRS-11 score and patient satisfaction at the postoperative follow-up (*p* < 0.001). Similarly, low NRS-11 scores at the postoperative follow-up increased physician satisfaction, and there was a moderate (*r*=−0.672) negative correlation between these parameters (*p* < 0.001) (Table 5).

**Table 5:** Correlation analysis of the likert scale

	*n*	Correlation coefficient(*r*) (95% CI)	*P* value
**Likert Scale (doctor) and Likert Scale (patient)**	50	0.857^b^(0.76 to 0.92)	**< 0.001**
**Likert Scale (patient) and NRS11 procedural**	50	−0.830^b^ (−0.90 to −0.72)	**< 0.001**
**Likert Scale (doctor) and NRS11 procedural**	50	−0.803^b^ (−0.88 to − 0.68)	**< 0.001**
**Likert Scale (patient) and NRS11 postprocedural**	50	−0.749^b^ (−0.86 to −0.61)	**< 0.001**
**Likert Scale (doctor) and NRS11 postprocedural**	50	− 0.672^a^ (−0.80 to −0.49)	**< 0.001**

Boldfaced *p*-values indicate statistically significant results

*CI* confidence interval

^a^moderately correlated, ^b^highly correlated

## Discussion

This study demonstrated that preoperative administration of RIB in patients undergoing CIED implantation led to a reduction in NRS-11 scores during intraoperative and postoperative follow-up within the first 24 h. In the RIBG, LA requirements and postoperative analgesic consumption were found to be lower. Accordingly, the need for opioids was also reduced in this group. Additionally, higher levels of patient and physician satisfaction were observed in the RIBG, and a correlation was found between lower NRS-11 scores and increased patient satisfaction.

In anaesthesiology, the use of nerve or fascial plane blocks to achieve intraoperative anaesthesia and postoperative analgesia is becoming more prevalent. The use of fascial plane blocks to provide analgesia in cardiac surgery has increased in recent years [30, 31]. However, the literature on the application of fascial plane blocks in CIED implantations is limited.

The standard anaesthesia method in CIED implantation is conscious sedation with LA. However, this method is not always sufficient. The patient feels pain and may move during the procedure [32]. This method may lead to interruptions in the procedure, necessitating the administration of additional LA. Increasing the amount of LA administered may lead to problems such as delayed wound healing and local anaesthetic toxicity [33].

Our study analysed the outcomes of RIB application in CIED implantations. The primary reason for our preference for RIB was that the T2-T9 dermatomal intervals, where the RIB is effective, cover the region where the CIED is implanted. Another reason was that the block was applied between the rhomboid and intercostal muscles, which are easily accessible via ultrasonography. The RIB has additional advantages: it is performed with a single needle insertion. Moreover, the block can be performed with the patient in a sitting position, providing ease of application and comfort for the physician and the patient.

Maintaining sterile conditions during CIED implantation is crucial to minimize the risk of infection at the battery pocket and lead site. During RIB, a plane block is unnecessary around the battery insertion site. Thus, the risk of infection and hematoma is minimized.

A review of the literature regarding nerve blocks applied for CIED implantation revealed that predominantly anterior chest wall fascial plane blocks were included [34, 35]. In a study by Zafar et al., effective analgesia was provided for 24 h postoperatively, with pectoralis nerve block-II (PECS-II) applied in addition to LA in CIED implantations [36].

Arasu et al. compared patients who underwent pectoral nerve block-I (PECS-I) and patients in whom PECS-I and transverse thoracic muscle (TTM) blocks were simultaneously performed in CIED implantations. The need for postoperative opioids decreased in patients in whom the two blocks were applied together [34]. These researchers also stated that TTM is challenging to access because of its deep location and proximity to the pleural region, which makes the performance of the block difficult and necessitates clinician experienced in ultrasonography. In addition, the PECS-I block and TTM block require needle insertion through the patient’s anterior chest wall at two different locations. This approach causes the patient to feel more pain during the procedure. In addition, the PECS-I and TTM block areas and the implantation site of the CIED battery are very close. This approach may pose a risk for infection and hematoma.

Increasing the depth of sedation during CIED implantation may lead to complications such as respiratory depression. In addition, since the duration of action of local anaesthetics is usually short, patients may experience pain in the postoperative period after CIED implantation [35].

In the study by Bode et al., 60% of patients who underwent CIED implantation with LA had moderate to severe pain in the first 24 h after operation. These authors reported that 55% of patients who underwent CIED implantation experienced persistent pain in the first 6 h postoperatively. Pain that persisted for 8–24 h postoperatively was observed in 40% of patients [37]. In a large cohort study by Nair et al., the postoperative pain status of patients who underwent CIED was evaluated with the visual analogue scale (VAS). In this study, more than one-fourth of the patients experienced moderate pain (VAS score 4–6), whereas the same proportion of patients experienced severe pain (VAS score 7–10) [38]. In our study, we preferred NRS-11 scoring because it is an easy method. We found that the RIB decreased the NRS-11 score during both the intraoperative and postoperative periods. Creating the CIED pocket is one of the most painful steps during the implantation of the CIED. Consistent with this, in our study, the highest level of pain was observed during battery pocket creation in the LAG.

All patients (100%) who underwent implantation with LA required additional LA, whereas only 1 patient (4%) who underwent RIB required this procedure. Factors affecting the pharmacokinetics of the local anaesthetic agent administered may have caused this difference.

Studies have shown that minor surgeries can also cause chronic pain, as can major surgeries. Therefore, managing acute pain is important [39, 40]. The RIB alone does not provide surgical anaesthesia, but when combined with the LA, it provides optimal surgical conditions for the patient and physician. In our study, the RIB was highly effective in treating postoperative pain. However, postoperative pain assessments at 24 h were not conducted. For a comprehensive evaluation of chronic pain, longer-term follow-up and assessment of the patients are necessary.

Opioid use not only contributes to higher overall consumption but may also induce hyperalgesia in patients during the perioperative period [41].

Although CIED implantation is considered a minor surgical procedure, postoperative pain is encountered in a high proportion of patients. Markman et al. reported that 11% of patients needed opioid prescriptions in the postoperative period after CIED implantation [41]. In this study, there was no postoperative opioid requirement for RIBG.

Effective management of acute postoperative pain is crucial to avoid the transition to chronic pain. Multimodal analgesic therapies should be used if necessary. These options may include intravenously administered analgesic drugs and nerve or fascial plane blocks. Our study revealed that the need for postoperative first rescue analgesia was very low in RIBG patients, whereas there was no need for second rescue analgesia. Accordingly, RIBG significantly reduced the need for postoperative intravenous analgesics. Therefore, plane blocks such as RIB should be offered to patients with CIED implantations.

In recent years, the importance of improving postoperative pain has gradually increased. Poor management of postoperative pain has been shown to have clinically negative consequences [42]. Gan et al. reported that well-managed postoperative pain was associated with patient satisfaction [43]. Our study revealed a strong positive correlation between postoperative analgesia provided by RIB and patient satisfaction.

This study has several limitations. The first limitation is that the study was a single center study conducted with a small group of patients (n:50). Larger patient cohorts are required to conduct studies that will yield more robust and statistically significant results. Additionally, we acknowledge that the primary outcome (pain scores) was assessed at several different time points throughout the study, which necessitated the use of multiple statistical comparisons. While this approach may increase the risk of Type I error, the consistency of statistically significant differences observed across all time points suggests that the likelihood of false-positive findings is low. Nonetheless, this should be considered a potential limitation of our study. The RIB procedure for the intervention group was performed in a separate room 60 min prior to the patient’s transfer to the cardiac electrophysiology laboratory, as described in the methodology section. When the patient arrived at the electrophysiology lab, the cardiologist performing the CIED implantation was not informed whether RIB had been administered beforehand. Cardiologist was blinded to the treatment. However, due to the nature of the study, patients were not blinded to the treatment. The third limitation is that the follow-up period was limited to the first 24 h after operation. There were no postdischarge follow-up data; therefore, it is not known whether the patients experienced chronic pain. During the follow-up of CIED patients, it is important for cardiologists to consider screening for chronic pain symptoms. Patients who are suitable for referral may be directed to specialists to improve their comfort.

## Conclusion

In conclusion, the rhomboid intercostal plane block provided effective analgesia in patients undergoing cardiac implantable electronic device implantation. Its administration significantly reduced the requirement for additional local anaesthetic and rescue analgesics during the perioperative period. Furthermore, it was associated with increased satisfaction among both patients and cardiologists. It is recommended that anaesthesiologists and cardiologists collaborate to develop standardised protocols incorporating rhomboid intercostal plane block as part of multimodal perioperative analgesia strategies in cardiac implantable electronic device implantations.

## Data Availability

The datasets used and/or analyzed during the current study are available from the corresponding author on reasonable request.

## Abbreviations

AF: Atrial Fibrillation
ASA: American Society of Anesthesiologists
AT: Auscultation Triangle
BMI: Body Mass Index
CAD: Coronary Artery Disease
CICU: Coronary Intensive Care Unit
CIED: Cardiac implantable electronic device
CKD: Chronic Kidney Disease
CP: Cardiac Pacemaker
CRT: Cardiac Resynchronization Therapy
CS: Conscious Sedation
HCL: Hydrochloride
ICD: Implantable Cardioverter Defibrillator
Impl.: Implantation
IV: Intravenous
LA: Local Anaesthesia
LAG: Local Anaesthesia Group
Min: Minutes
Ml: Millilitre
NRS-11: Numeric Rating Scale-11
NYHA: New York Heart Association
PNB-I: Pectoral nerve block-I
PNB-II: Pectoral Nerve Block-II
RIB: Rhomboid Intercostal Plane Block
RIBG: Rhomboid Intercostal Plane Block Group
SD: Standard Deviation
TTM: Transverse Thoracic Muscle
USG: Ultrasound
VAS: Visual Analogue Scale
